# Meningeal carcinomatosis secondary to neurenteric cysts with malignant transformation: a case report

**DOI:** 10.1186/s12883-022-02978-7

**Published:** 2022-11-16

**Authors:** Min Chu, Leiming Wang, Hong Ye, Junjie Li, Dehong Lu, Yueshan Piao, Liyong Wu

**Affiliations:** 1 Department of Neurology, Xuanwu Hospital, Capital Medical University, Beijing, 100053 China; 2 Department of Neuropathology, Xuanwu Hospital, Capital Medical University, Beijing, 100053 China

**Keywords:** Meningeal carcinomatosis, Neurenteric cysts, Case report

## Abstract

**Background:**

Meningeal carcinomatosis is mainly associated with breast cancer, lung cancer, and melanoma. However, meningeal carcinomatosis secondary to a neurenteric cyst with malignant features is extremely rare.

**Case presentation:**

We report the case of a 35-year-old woman who was admitted to the hospital with a 10-month history of headache, 6-month history of diplopia, 4-month history of hearing loss, and 1-month history of back pain, suggesting a diagnosis of chronic meningitis. Notably, enhanced brain and spinal cord magnetic resonance imaging (MRI) revealed extensive lesions with enhancement signals in the pia mater of the pons and cervical, thoracic, and lumbar spinal cord. The cerebral spinal fluid profile showed that pressure was significantly elevated, with a slight increase in leukocytes that mostly comprised mononuclear cells and decreased glucose concentration. Cytology evaluation showed a small cluster of atypical nuclei, which were suspected to be tumor cells arising from the epithelium. However, no primary tumor was found through comprehensive body and skin screening. After a histopathological biopsy of subarachnoid meninx of the thoracic spinal canal, the cause of meningeal carcinomatosis of this patient was determined as neurenteric cysts with malignant features, which is extremely rare.

**Conclusion:**

This is the first case to ever report neurenteric cysts as a cause of leptomeningeal carcinomatosis and the first ever report of neurenteric cysts presenting as leptomeningeal carcinomatosis without typical cyst visible on brain MRI. This extremely rare case provided a novel view on the pathogenesis of meningeal carcinomatosis and clinical presentation of neurenteric cysts, highlighting the value of meningeal biopsy in chronic meningitis of unknown causes.

## Background

Meningeal carcinomatosis (MC, also known as leptomeningeal metastasis, neoplastic meningitis, carcinomatous meningitis, and leptomeningeal carcinomatosis) is an end-stage complication of cancer with poor prognosis and nonspecific symptomatic presentation characterized by the seeding of metastatic malignant cells on the leptomeninges [1]. Meningeal carcinomatosis is mainly associated with breast cancer, lung cancer, and melanoma [2–4]. However, meningeal carcinomatosis secondary to neurenteric cysts with malignant features is rare.

## Case presentation

A 35-year-old woman was admitted to the hospital with a 10-month history of headache, 6-month history of diplopia, 4-month history of hearing loss, and 1-month history of back pain. No fever, tumors, or exposure history of specific infections were noted. No significant family history of malignancy was found. Neurologic examination revealed normal cognitive performance, limb motor function, reflexes, sensory and cerebellar functions. Difficulty in bilateral eyeball abduction, diplopia, and mild bilateral facial paralysis were noted. There was hearing loss in both ears by gross hearing assessment. Signs of meningeal irritation, including Brudzinski and Kernig signs, were positive, and nuchal rigidity was present.

**Fig. 1:**
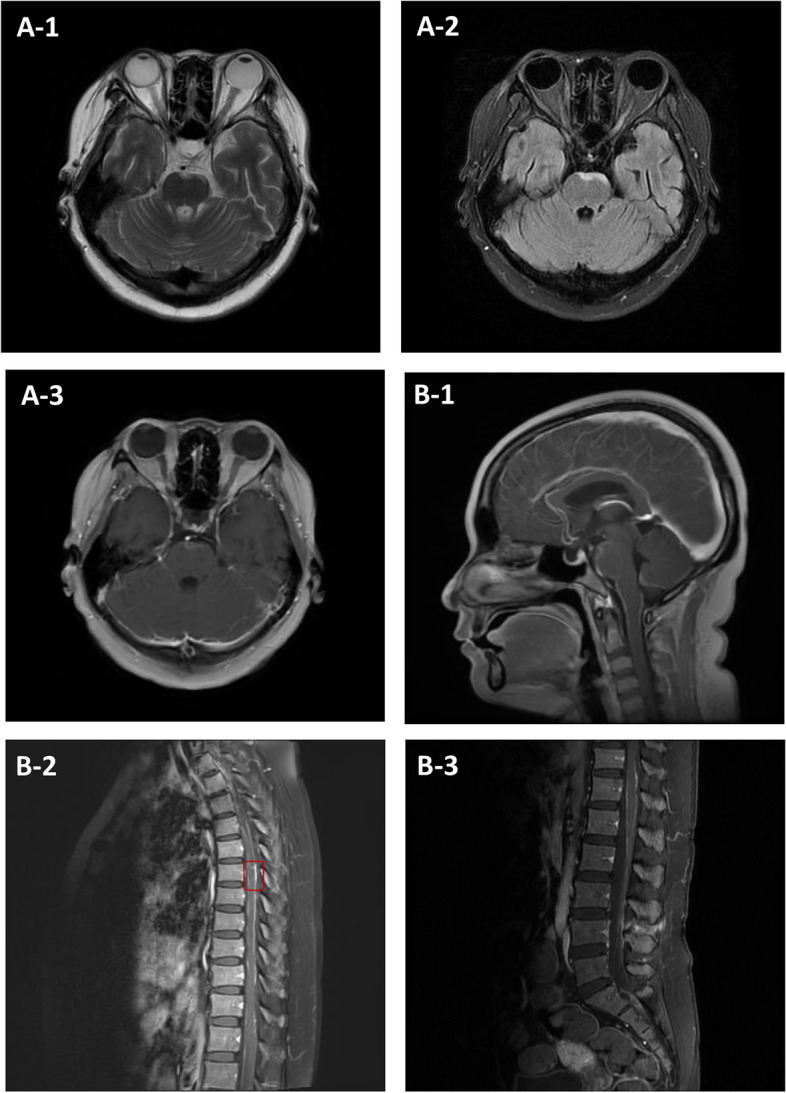
MRI findings**. A-1.** Axial MRI showing stripe hyperintensity in the pontine base on T2 sequence. **A-2.** Axial MRI showing stripe hyperintensity in the pontine base on FLAIR sequence. **A-3.** Faint enhancement of the abnormal pontine lesion. **B-1**. Sagittal contrast-enhanced MRI showing enhanced signal in the pia mater of the pontine and upper cervical spinal cord. **B-2** and **B-3**. Sagittal contrast-enhanced MRI showing enhanced signal in the pia mater of the spinal cord (T1–T12 and L1–L2 levels). The site of the biopsy specimen of the subarachnoid meninx of the thoracic spinal canal is marked in the red box

**Fig. 2:**
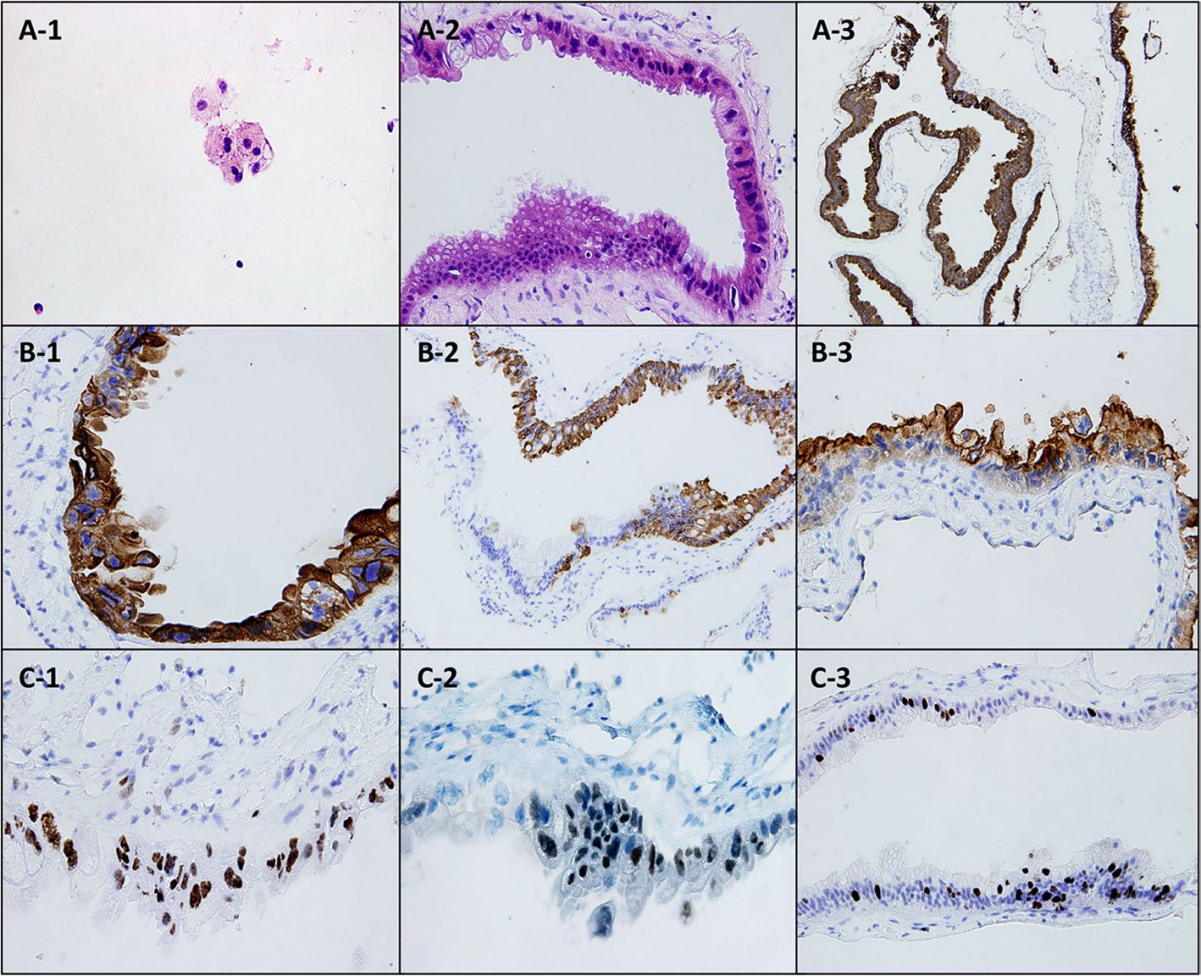
Histologic examination. **A-1**. Atypical nuclei are noted in CSF cytology. **A-2**. Fibrocystic wall-like tissue, covered by columnar epithelium and mucus epithelium, with partial malignant characteristics. **A-3, B, C**. Immunostaining results. **A-3.** CK positive. **B-1.** CK7 positive. **B-2.** Partially CK20 positive. **B-3.** CEA positive. **C-1.** P53 positive. **C-2.** Partially CDX-2 positive. **C-3.** Ki-67 staining shows a proliferation rate of 20%. Abbreviations: CEA, carcinoembryonic antigen; CK, cytokeratin

Cerebrospinal fluid (CSF) pressure was significantly elevated, with a slight increase in leukocytes that mostly comprised mononuclear cells and decreased glucose concentration. No pathogenic microorganisms were found by next-generation sequencing. Enhanced brain magnetic resonance imaging (MRI) showed an abnormal signal on the ventral side of the pons (Fig. 1 A-1 and A-2) and enhancement (Fig. 1 B-1). Enhancement was also observed in the thickened dura of the upper cervical spinal cord (Fig. 1 B-2). MRI of the thoracic (Fig. 1 C-1) and lumbar (Fig. 1 C-2) spinal cord showed thickening and enhanced spinal pia mater signals at the thoracic 1–12 and lumbar 1–2 levels.

Considering the possibility of meningeal carcinomatosis, an examination of CSF was conducted (Fig. 2 A-1), in which small clusters of atypical nuclei were noted, which were suspected to be tumor cells. Further immunohistochemical special staining results were CK (-), CK7( +), ki-67(-), and AB/PAS ( +), suggesting that the origin of the tumor cells might be the epithelium. No tumors were found on systemic positron emission tomography-computed tomography (PET–CT). No cutaneous melanocytic nevi were found in a careful examination of the patient’s skin. Histological examination of the subarachnoid meninx of the thoracic spinal canal in thoracic 6 level was performed. The specimen (Fig. 2 A-2) showed a cystic lesion covered by simple and pseudostratified, ciliated columnar epithelium, which was rich in mucin-producing cells highlighted by Alcian blue staining. Malignant characteristics were observed in some areas, including increased cellular density and pleomorphism with hyperchromatic nuclei. Immunostaining (Fig. 2 A-3, B1–B3, and C1-C3) showed that the cells were positive for epithelial membrane antigen, cytokeratin, and carcinoembryonic antigen but negative for glial fibrillary acidic protein (GFAP), thyroid transcription factor (TTF)-1, and cancer antigen (CA)125. An increased proliferation labeling index (MIB-1, Ki-67) was observed in the areas of malignant transformation (about 20% of nuclei). A diagnosis of the neurenteric cyst with focal malignant features was considered. Malignant Rathke's cleft cyst was considered as a differential diagnosis; however, there was no granulomatous change or squamous metaplasia in the patient. Malignant endolymphatic sac tumor was also considered as a differential diagnosis; however, no petrous bone abnormalities were noted.

The patient refused radiation treatment and intrathecal or systematic chemotherapy and selected palliative treatment with ventricular abdominal drainage. After the ventricular abdominal drainage surgery, the headache was alleviated, but other symptoms still persisted. Until we submitted the case report, she was bedridden and in an extremely weakened state.

## Discussion and conclusions

Neurenteric cysts are congenital anomalies that are thought to result from a failure of the neuroectoderm and endoderm to separate in the third week of embryogenesis. They are typically located on the ventral side of the cervical or thoracic spinal cord [5]. The neurenteric cysts with malignant features mainly occur in adult patients aged between 20 and 50 years [6]. The most typical neurenteric cysts reported in the literature are ovoid/lobulated hyperintense masses in front of the medulla [5]. Additionally, approximately 17.9% of the neurenteric cysts are intracranial, and most are located in the posterior fossa, usually anterior to the brainstem or in the cerebellopontine angle [5, 7].

The signal intensity features of intraspinal and intracranial cysts vary depending on the protein content of the cyst fluid [8, 9]. Typical neurenteric cysts often appear hyperintense or isointense on T1-weighted images and hyperintense on T2-weighted images, occasionally with enhancement or partial enhancement on Gd-enhanced MRI scans [8, 10]. However, no patient with intracranial neurenteric cysts has been reported to date with extensive meningeal carcinomatosis and without actual cysts.

A diagnosis of a neurenteric cyst depends on histopathology. The cyst lining observed by microscopy can range from simple to pseudostratified low cuboidal or columnar epithelium, with or without cilia [5]. The pathological features of our patient were typical and consistent with the literature. In addition, we found malignant features in the biopsy specimen, which was extremely rare. To our knowledge, only nine cases have been previously reported [6, 11, 12].

Symptoms and signs of meningeal carcinomatosis mainly depend on the site of invasion, manifested as general symptoms (e.g., headache, mental status changes, confusion, and seizures), cranial nerve involvement (VI, VII, and VIII are commonly affected, leading to diplopia, facial palsy, and hearing loss, respectively), spinal nerve dysfunction (radicular pain, sensory loss, bowel and bladder dysfunction, and limb weakness), and meningeal irritation [1]. Increased intracranial pressure (ICP) and hydrocephalus may be observed as the metastatic carcinoma obstructs the CSF outflow. Symptoms tend to worsen with disease progression. Our patient had cranial nerve VI, VII, and VIII involvement due to the invasion of malignant neurenteric cysts into the anterior ventral pons. Its dissemination resulted in extensive meningeal involvement, leading to severe headache, papilledema, and meningeal irritation.

Most patients with meningeal carcinomatosis have no brain parenchymal metastases: tumor cells mostly infiltrate diffusely into the meninges and subarachnoid space. CT findings may be normal, frequently resulting in misdiagnosis. Gadolinium-enhanced T1-weighted MRI of the brain and spine is recommended if there is clinical suspicion, irregular lesion signals, and linear or nodular leptomeningeal enhancement [13]. Our patient showed an abnormal-stripe hyperintensity in the anterior ventral pontine on MRI, with thickening and enhancement of the upper cervical, thoracic, and lumbar pia mater, but no obvious tumors in the brain parenchyma, which was in line with the typical imaging characteristics of meningeal carcinomatosis.

Lumbar puncture is recommended if it can be safely performed. Suggestive abnormalities include increased opening pressure (> 200 mm H_2_O), increased leukocytes (> 4/mm^3^), elevated protein concentration (> 50 mg/dl), and decreased glucose level (< 60 mg/dl) [14]. A definitive diagnosis depends on CSF cytology or flow cytometry [15, 16] and may be facilitated by CSF tumor markers and circulating tumor deoxyribonucleic acid (DNA) [17, 18]. Therapy for neurenteric cysts with malignant features involves a multimodal approach focused on surgery, radiation, and intrathecal or systematic chemotherapy [6].

According to the time sequence of clinical symptoms, we speculated that the primary neurenteric cysts lesion might be at the pons, then spreading through the arachnoid membrane and leading to extensive cerebrospinal membrane involvement. The limitation of this clinical case report was that we did not conduct a direct biopsy of the pons to confirm the diagnosis because of the high risk for biopsy in this significant brain region.

In conclusion, we have reported an extremely rare case that manifested as meningeal carcinomatosis and was diagnosed with pathologically-proven neurenteric cysts with malignant transformation. However, no obvious actual cyst was observed on MRI, which was different from the cases of previously reported neurenteric cysts. This extremely rare case provided a novel view on the pathogenesis of meningeal carcinomatosis and clinical presentation of neurenteric cysts. For chronic meningitis without definite infection or evidence of tumor, meningeal biopsies should be performed if necessary to identify the diagnosis.

## Data Availability

The datasets used and analyzed during the current study are available from the corresponding author upon reasonable request.

## Abbreviations

MRI: Magnetic resonance imaging
MC: Meningeal carcinomatosis
CSF: Cerebrospinal fluid
PET-CT: Positron emission tomography-computed tomography
GFAP: Glial fibrillary acidic protein
TTF: Thyroid transcription factor
CA: Cancer antigen
CT: Computed tomography
DNA: Deoxyribonucleic acid
